# Supplementary material for: Heterogeneous Ribonucleoprotein K Is a Host Regulatory Factor of Chikungunya Virus Replication in Astrocytes
Source: Viruses. 2024 Dec 14;16(12):1918. doi: 10.3390/v16121918 (PMC11680317; doi:10.3390/v16121918)
Supplement: Supplementary file 1 [file viruses-16-01918-s001.zip › viruses-3342387-supplementary.pdf]

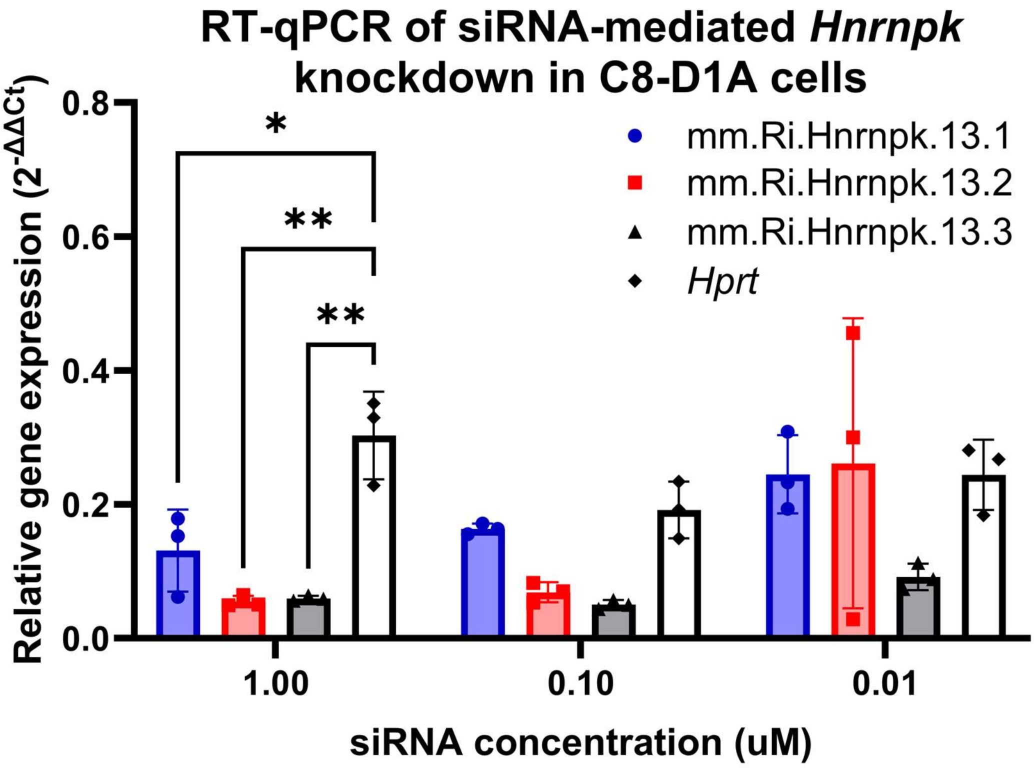

Figure S1. Small interfering RNA (siRNA)-mediated knockdown of *Hnrnpk* was verified via RT-qPCR analysis using RNA lysates collected from murine astrocytes (C8D1A) transfected for 48 hours. Cycle threshold values were normalized to *Gapdh*-VIC levels. Experimental design included the use of three biological replicates per condition. Individual replicate values are denoted by symbols delineated by the legend, whilst standard deviations of the mean are represented by error bars. Statistical significance was determined via two-way ANOVAs, assuming Gaussian distribution; *, $P < 0.05$; **, $P < 0.01$.
